# Micro-CT imaging and finite element models reveal how sintering temperature affects the microstructure and strength of bioactive glass-derived scaffolds

**DOI:** 10.1038/s41598-023-50255-5

**Published:** 2024-01-10

**Authors:** Anna De Cet, Luca D’Andrea, Dario Gastaldi, Francesco Baino, Enrica Verné, Gissur Örlygsson, Pasquale Vena

**Affiliations:** 1 Department of Chemistry, Materials and Chemical Engineering “Giulio Natta”, Laboratory of Biological Structure Mechanics (LaBS)-Politecnico di Milano, Piazza Leonardo da Vinci 32, 20133 Milan, Italy; 2 Institute of Materials Physics and Engineering, Department of Applied Science and Technology-Politecnico di Torino, 10129 Turin, Italy; 3 IceTec, 112 Reykjavik, Iceland

**Keywords:** Biomedical engineering, Implants

## Abstract

This study focuses on the finite element simulation and micromechanical characterization of bioactive glass-ceramic scaffolds using Computed micro Tomography ($\upmu$CT) imaging. The main purpose of this work is to quantify the effect of sintering temperature on the morphometry and mechanical performance of the scaffolds. In particular, the scaffolds were produced using a novel bioactive glass material (47.5B) through foam replication, applying six different sintering temperatures. Through $\upmu$CT imaging, detailed three-dimensional images of the scaffold’s internal structure are obtained, enabling the extraction of important geometric features and how these features change with sintering temperature. A finite element model is then developed based on the $\upmu$CT images to simulate the fracture process under uniaxial compression loading. The model incorporates scaffold heterogeneity and material properties—also depending on sintering temperature—to capture the mechanical response, including crack initiation, propagation, and failure. Scaffolds sintered at temperatures equal to or higher than 700 $^{\circ }$C exhibit two-scale porosity, with micro and macro pores. Finite element analyses revealed that the dual porosity significantly affects fracture mechanisms, as micro-pores attract cracks and weaken strength. Interestingly, scaffolds sintered at high temperatures, the overall strength of which is higher due to greater intrinsic strength, showed lower normalized strength compared to low-temperature scaffolds. By using a combined strategy of finite element simulation and $\upmu$CT-based characterization, bioactive glass-ceramic scaffolds can be optimized for bone tissue engineering applications by learning more about their micromechanical characteristics and fracture response.

## Introduction

This paper deals with Bone Tissue Engineering (BTE) glass-derived scaffolds for the treatment of critical size bone defects^1–4^. In order to ensure the correct functionality and biocompatibility of BTE scaffolds, structural, mechanical and biological requirements should be met^3,5^. Particularly, the elastic modulus should match those of the tissue surrounding the implantation site to minimize stress shielding and implantation-related osteopenia^6,7^ and, simultaneously, guarantee a sufficient strength^8^, depending on the anatomical site of the implant.

Experimental mechanical tests are extensively used to assess the stiffness and the strength of scaffolds, which are strictly related to the bulk materials and manufacturing techniques^9–12^. However, they provide macroscopic properties which cannot be directly correlated with the scaffold architecture or with the intrinsic properties of the constituent materials. Only a backcasting analysis can be performed on the broken samples to speculate on the role of microarchitecture on crack propagation and ultimately on the scaffold strength. In-situ mechanical testing combined with concurrent $\upmu$CT scanning is a sophisticated method for examining the mechanical behavior of intricate 3D microstructures. This approach enables precise analysis of the complex 3D pattern of damage or fracture through the use of Digital Volume Correlation. This technique has been successfully applied to trabecular bone^13^, cortical bone^14^ and polymer scaffolds reinforced with ceramic particles^15,16^. Since ceramic scaffolds are brittle, it is challenging to examine the scaffolds at intermediate configurations before a substantial fracture had propagated, especially in highly porous scaffolds. This methodology is applied to fully ceramic scaffolds in one work^17^ for hydroxyapatite scaffolds, reporting few time steps of the loading process.

Validated $\upmu$CT-based finite element models (FEM) replicating scaffold properties^18–21^ can disclose the role of the microstructure in determining the macroscopic properties of the scaffolds. This can be achieved by simulating the fracture propagation process and quantitatively estimating the elasticity and strength allowing for geometrical imperfections, intrinsic porosity and intrinsic mechanical properties of the constituents deriving from the specific manufacturing process^18,22^. This methodology can substantially reduce the costs associated with the design and development of optimized devices.

Bioactive glass materials have been widely used as constituent materials for bone tissue engineering scaffolds^23,24^, as they are able to bond strongly with the surrounding tissue and support osteogenesis since they exhibit osteoconductive and osteoinductive properties^25^.

Foam replication is a production technique based on particle sintering, which uses sacrificial templates to obtain highly porous and interconnected scaffolds with a controllable pore size^20,26–29^. The sintering temperature of the scaffold plays a key role in the microstructure arrangement of the solid fraction of the scaffold^30^ but its impact on the mechanical properties of the scaffold is still unknown.

The scaffold geometries analyzed in this work were obtained from $\upmu$CT-scans of foam-replicated products based on 47.5B glass^31,32^. The main aim was to estimate the effect of the sintering temperature on the morphometrical properties and on the mechanical properties of the produced scaffold by means of $\upmu$CT-based FEM. A partial validation of the mechanical properties was done by comparing the in silico strength values with the experimental values present in the literature^32^. Furthermore, the fracture mechanisms and patterns have been addressed and related to the morphometric features of the scaffolds. These unique pieces of information represent a turning point for the design and manufacturing of suitable scaffolds for BTE applications toward the future development of tailored and personalized devices for critical-size bone defects.

## Results

### Morphological characterization

The morphological characterization has shown that sintering temperature has a substantial impact on the micro-architecture of the resulting scaffolds. On the basis of the results presented in^31^, two groups of scaffolds are defined: one group for scaffolds sintered at 650 $^\circ$C or lower, which from now on will be denoted as low temperature (LT) scaffolds, and one group sintered at 700 $^\circ$C or higher, here denoted as high temperature (HT) scaffolds. The LT scaffolds are sintered at a temperature below the crystallization temperature ($T_c$); while the HT scaffolds are sintered above $T_c$.

**Figure 1:**
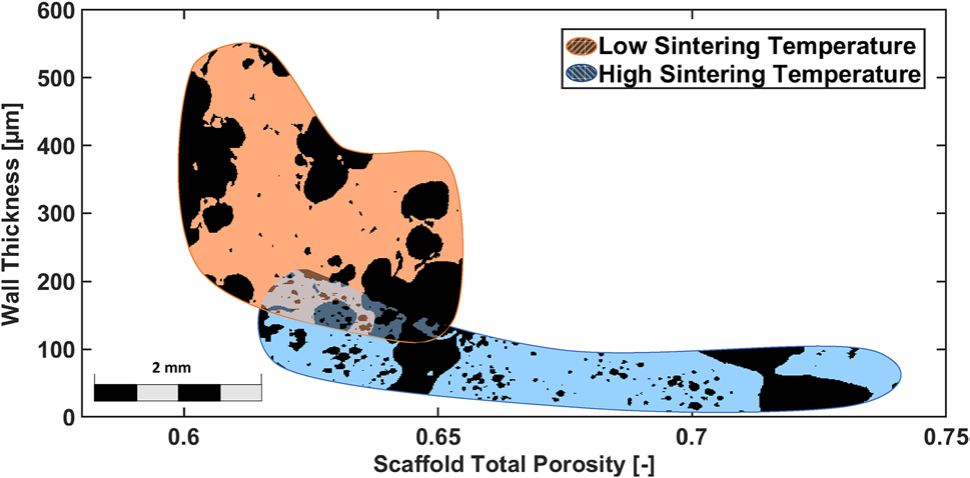
Wall thickness versus total porosity. The two coloured regions are obtained as the envelope of the mean value and standard deviation of the wall thickness as computed by BoneJ for HT and LT scaffolds. More specifically, the envelopes have been built utilizing one value of porosity per scaffold (and therefore per temperature) on the horizontal axis, while the corresponding value of wall thickness is reported on the vertical axis as mean value and standard deviation. Inside each region, slices of the binarized $\upmu$CT visualise the different micro-architectures for the HT and LT scaffold groups. Particularly, the black part of the images correspond to the voxels of the 3D scaffolds which are empty (i.e. the pores), while the colored regions correspond to the solid parts of the scaffolds. The two different colours were chosen as representative of the LT and HT groups.

The average scaffold porosity values are around 60% in LT scaffolds, while HT scaffolds display an average porosity varying between 60 and 80%. As shown in both Fig. 1 and Table 1 the total porosity was found to be changing depending on sintering temperature, but no clear trend could be observed for these specific samples. The difference in porosity found between scaffolds belonging to the LT group and the HT group is consistent with what was found in a previous study^31^.

**Table 1:** Predicted macroscopic strength for two possible average flaw size: $10\;\upmu$m and $20\;\upmu$m.

Sintering temperature [${}^\circ$C]		Porosity [-]	Scaffold compressive strength		
			$\sigma \;/\;\sigma _0$ [-]	$\sigma ^{10}$ [MPa]	$\sigma ^{20}$ [MPa]
LT	600	64%	0.05	5.13	3.67
	650	60%	0.06	6.93	4.93
HT	700	74%	0.02	3.21	2.29
	750	71%	0.03	4.16	2.93
	800	69%	0.03	5.80	4.11
	850	60%	0.04	8.85	6.31

Figure 1 shows the spread of the wall thickness in relation to the total scaffold porosity. The wall thickness ranges between 100 and $500\;\upmu$m in the LT scaffolds, while in HT scaffolds it varies between 10 and $200\;\upmu$m. In order to provide a visual representation of the difference in micro-architecture between HT and LT scaffolds, two representative sections of slices of the $\upmu$CT images of the scaffolds have been inserted in Fig. 1; the black portions of the images correspond to the empty space (i.e. the pores), while the coloured parts (orange for LT scaffolds and light blue for HT scaffolds) represent the solid portions of the scaffolds. In particular, $\upmu$CT images show the formation of a double porosity system in HT scaffolds as specified below.

**Figure 2:**
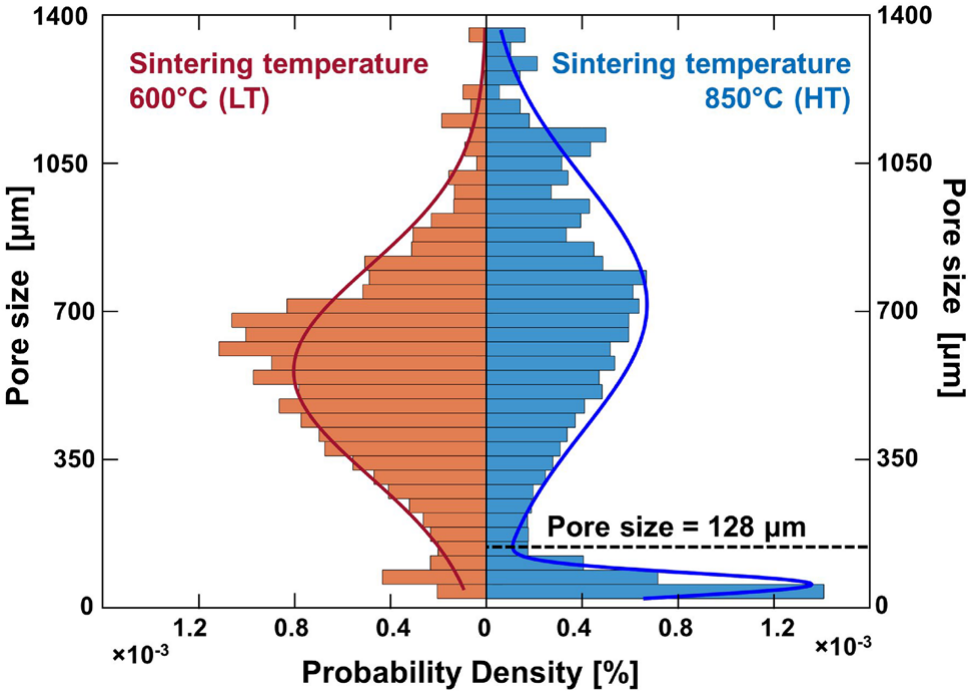
Pore size distribution two representative temperatures: 600 $^{\circ }$C for the LT group and 850 $^{\circ }$C for HT group.

Figure 2 shows the histograms of pore size for two representative sintering temperatures: $600\,^\circ$C and $850\,^\circ$C. While the LT exhibits a single-peak pore-size distribution, the HT show a two-peaks distribution. This indicates that at high sintering temperatures, two groups of porosity are formed with two different characteristic sizes: larger pores are approximately $600\;\upmu$m and smaller pores are in the order of $100\;\upmu$m. The threshold value separating the two groups of pores is $128\;\upmu$m.

### Macroscopic elastic modulus of scaffolds

The elastic moduli estimated through the $\upmu$CT-based finite element analyses are reported in Table 2. In particular, both the elastic modulus normalized with respect to the intrinsic elastic modulus of the constituent materials (specified for each sintering temperature) and the macroscopic elastic moduli are reported.

**Table 2:** Elastic moduli normalized with respect to intrinsic elastic modulus and macroscopic elastic modulus along the longitudinal direction for the cylindrical samples; the values are based on finite element analysis.

Sintering temperature [${}^\circ$ C]	Normalized elastic modulus [-]	Scaffold elastic modulus [GPa]
LT		
600	0.208	16.65
650	0.306	26.01
HT		
700	0.085	8.60
750	0.117	12.08
800	0.030	2.43
850	0.118	12.01

By grouping the scaffolds as done for the morphology in LT and HT scaffolds the average normalized elastic modulus was 0.26 and 0.09 for LT and HT scaffolds, respectively. The macroscopic elastic moduli were 21.33 GPa and 8.78 GPa for the LT and HT scaffolds, respectively. These results indicate that the effect of temperature on the micro-architecture has also a substantial effect on the elastic mechanical properties of porous scaffolds.

### Strength analyses

**Figure 3:**
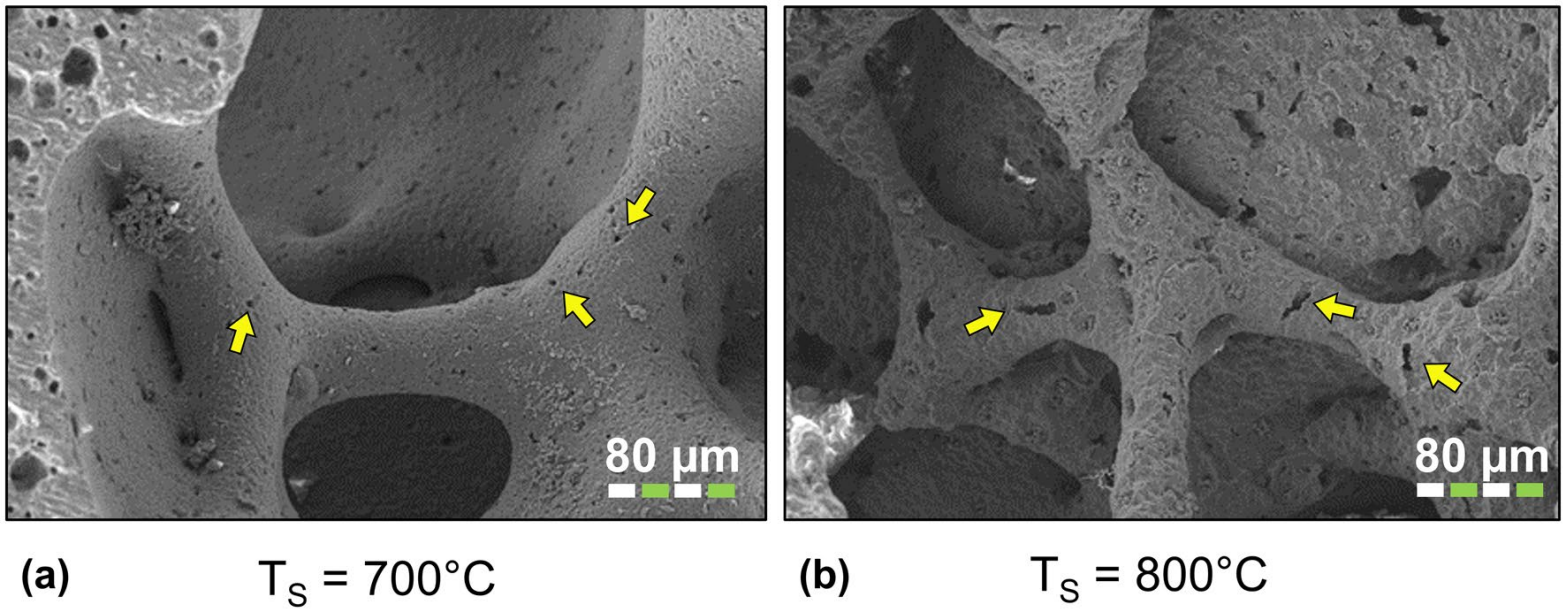
Two representative SEM images: (**a**) $T_S = 700\,^{\circ }$C and (**b**) $T_S = 800\,^{\circ }$C. The yellow arrows point to typical flaws with characteristic size up to $20\;\upmu$m.

Table 1 shows the normalized compressive strength $\sigma /\sigma _0$ and two different values of the macroscopic compressive strength as obtained by means of the $\upmu$CT finite element simulation of an uniaxial compressive load, $\sigma ^{10}$ and $\sigma ^{20}$. The two latter compressive strengths have been obtained by setting the intrinsic strength of the constituent material at each sintering temperature as found in Ref.^33^ by using two different values of intrinsic defect, namely $10\;\upmu$m and $20\;\upmu$m, respectively (Fig. 3).

Furthermore, the porosity of each scaffold is reported to highlight the correlation between this morphological parameter and the mechanical property of each sample.

The average normalized compressive strength was 0.06 and 0.03 for LT and HT scaffolds, respectively. Similarly to the elastic properties, also normalized compressive strength is substantially affected by the different microstructure induced by the different sintering temperatures. However, average $\sigma ^{10}$ strength was 6.03 MPa and 5.5 MPa for LT and HT samples respectively. The mismatch between LT and HT on macroscopic strength is milder with respect to the normalized strength as the intrinsic strength of constituent material for HT was higher than the one found for LT scaffolds^33^.

**Figure 4:**
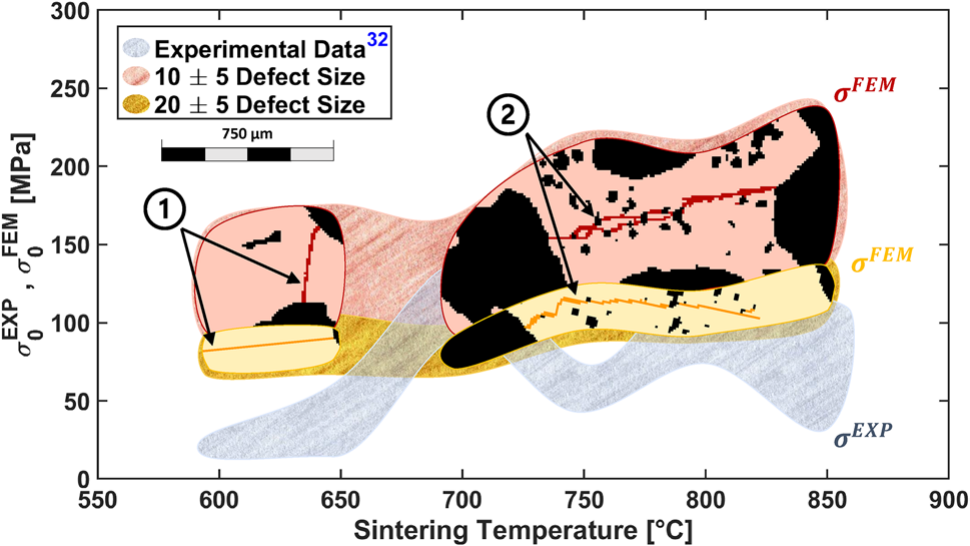
$\sigma _{0}^{FEM}$ and $\sigma _{0}^{EXP}$ versus sintering temperature. Two estimates of $\sigma _{0}^{FEM}$ are reported, for two values of intrinsic defects ($10\;\upmu$m and $20\;\upmu$m). Arrows indicate the two different fracture types identified through the Finite Element simulations: the fractures connecting large pores (1), mainly occurring in LT scaffolds and fractures connecting small pores (2) mainly occurring in HT scaffolds. Experimental data obtained in Ref.^32^ are used to determine $\sigma _{0}^{EXP}$.

Figure 4 shows a comparison between the compressive strength predicted by the finite element simulations and the available experimental strength at all sintering temperatures. Since the samples that have been tested and those that have been numerically simulated do not have the same porosity, the formula by Gibson and Ashby^34^ has been used to normalize strength with porosity for the comparison of the experiments and the numerical simulations reported in Fig. 4.

**Figure 5:**
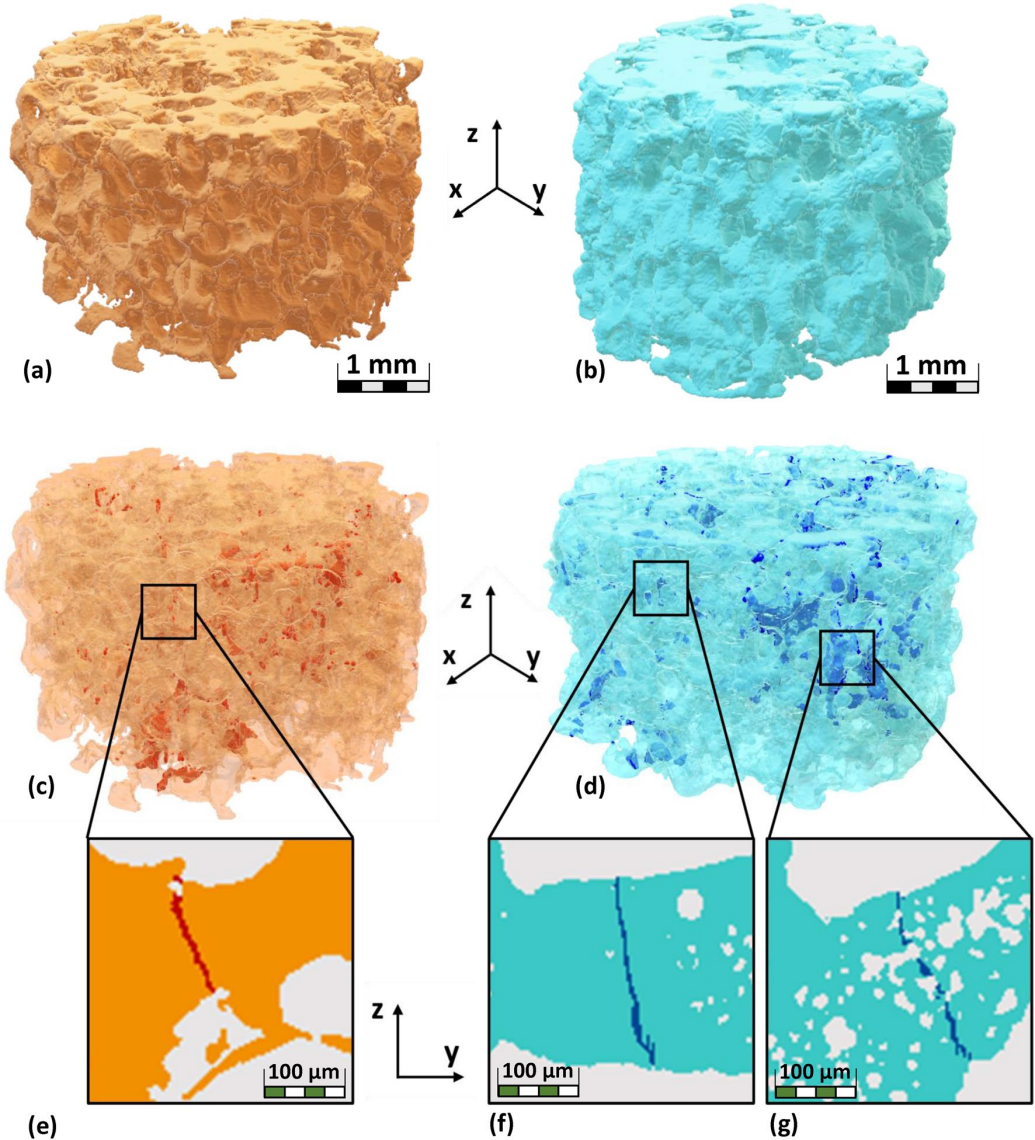
(**a**) $\upmu$CT reconstruction of a LT scaffold; (**b**) $\upmu$CT reconstruction of a HT scaffold; (**c**) LT scaffold highlighting the fractures due to compressive load; (**d**) HT scaffold highlighting the fractures due to compressive load; (**e**) fracture connecting large pores in LT scaffold; (**f**) fracture connecting large pores in HT scaffold; (**g**) fractures connecting small pores in HT scaffold.

The results are grouped in three major areas: (1) the group for average intrinsic material defect of $10\;\upmu$m; (2) the group for average intrinsic material defect of $20\;\upmu$m; and (3) the third group (in grey) represents the spread of the experimental values reported by Fiume et al.^31^. The selected ranges of defect sizes have been chosen in relation to the resolution of the $\upmu$CT images of the scaffolds after they have been pre-processed; one group is characterized by an average defect size that is equal to and lower than the image resolution, the other by larger defects. In both cases, the scaffold strength slightly increases with the sintering temperature. Furthermore, Fig. 4 reports the separation in the two groups of sintering temperatures (HT and LT) for each main defect size. In each respective area, images of the microstructure and of the fracture patterns obtained in the scaffolds are reported. In both cases, fractures originate and end in macroscopic pores, if micro-pores are present close to the fracture, fracture paths intersect the micro-pores. Figure 5 shows the fracture patterns of two representative samples ($600\,^\circ$C on the left panels and $850\,^\circ$C on the right panels). Figure 5 displays a broad overview of the scaffolds and their fracture pattern, found in samples that were subjected to compression loads as determined by finite element simulations.

Figure 5 demonstrates how the boundaries of the pores have an impact on the fracture pattern. The majority of the fracture patterns in samples with few and large pores (corresponding to a low sintering temperature) are vertical lines connecting these pores, whereas, in samples with many and small pores (corresponding to a high sintering temperature), the fracture paths are more erratic and cross micro-pores.

An evaluation of the solid volume involved in the fracture process during the uniaxial compression test (i.e. the volume of the elements deleted in the fracture propagation simulation) is carried out by processing the $\upmu$CT scan images and labeling each deleted element with the corresponding strut thickness.

The distribution of broken elements for the LT scaffold (sintered at $600\,^\circ$C) is evenly spread on the whole span of strut thickness. This indicates that struts tend to break with a comparable chance in regards to strut thickness.

On the other hand, only two types of struts in HT scaffolds are more likely to fracture: (a) those with a thickness lower than $100\;\upmu$m and (b) those with a strut thickness larger than $430\;\upmu$m. For case (a), this indicates that the fracture will impact the thin walls that divide the micro-pores that are found in HT scaffolds and link micro-pores that formed into clusters (refer to the focus images of Fig. 4 labeled with $\textcircled {2}$ and the planar slice for HT samples in Fig. 5g). A connection level of $\chi =73 \%$ is reached by the volume occupied by the isolated micro-pores, suggesting that many micro-pores in HT scaffolds will be connected by fractures. In contrast, in case (b) there are fractures that happen in thicker walls (i.e. larger than $430\;\upmu$m); these fractures link large pores or isolated small pores.

## Discussion

The purpose of this study was to describe the morphological and mechanical characteristics of glass-derived scaffolds made through foam replication and how the sintering temperatures affect these characteristics. The finite element modeling technique based on $\upmu$CT was chosen for this purpose.

The $\upmu$CT image analysis showed that sintering temperature has a substantial effect on the scaffolds’ micro-architecture. In particular, the scaffolds sintered at $650\,^{\circ }$C or lower (LT scaffolds) showed large pores separated by a solid matrix with an average wall thickness of $300\;\upmu$m; in contrast, the scaffolds sintered at $700\,^{\circ }$C or higher (HT scaffolds) showed pores with two distinct pore sizes, i.e. large pores separated by a matrix with small scale porosity. This finding is consistent with the analyses carried out by Fiume et al.^32^ in which the crystallization temperature of $690\,^{\circ }$C was identified as a critical temperature above which the microstructure of the scaffolds changes substantially.

The use of the computational method enables the evaluation of the effects of the scaffold structure and its inherent material properties on the mechanical behavior of the scaffolds as a whole.

The elastic modulus of the LT scaffolds is, on average, slightly higher than that found for HT scaffolds. The LT scaffold constituent material is however characterized by a lower elastic modulus; this highlights that the scaffold microstructure has a predominant influence on the overall scaffold elastic modulus.

Although the elastic moduli of these scaffolds have not been measured experimentally, the FEM method used to calculate them has been previously validated with estimates obtained using acoustic techniques for scaffolds made by foam replication^29^.

It is necessary to first assess the intrinsic strength of the materials that make up a scaffold before computing the scaffold strength. This is usually carried out through experimental tests on bulk samples of the constituent materials. The foam replication technique, however, does not enable the acquisition of samples with a shape suitable for conventional mechanical testing of small samples, such as micro-bending tests^35^. The strength analyses carried out in this work were fed with material properties (elastic modulus and tensile strength) obtained through nanoindentation^33^. In particular, the critical stress intensity factors were obtained for all sintering temperatures by measuring fracture length promoted by nanoindentation load. This provides only partial information, as the tensile strength of the material can be estimated only by making the hypothesis of a mode-I predominant fracture propagation mechanism and by estimating an intrinsic flaw size in the material owed to the manufacturing process. The flaw size that was assumed to evaluate the material strength is crucial for the quantitative prediction of the macroscopic strength of the scaffolds. Estimating the typical length of faults caused by the manufacturing process is a tough issue because, as Fig. 3 illustrates, these flaws may have a small width and a length of tens of microns, making it impossible to detect them using $\upmu$CT scans. Two representative flaw sizes, ($10\,\upmu$m and $20\,\upmu$m) are assumed in this work.

In a study by Entezari et al.^36^ $\upmu$CT-based FEM were used to determine the strength of ceramic scaffolds. Although those scaffolds’ porosity values are similar to those assessed for this study, their estimated strength is one order of magnitude higher. Furthermore, the tensile strength of the constituent material in the FEM models by Entezari et al. is 97 MPa, while the intrinsic tensile strength of the LT and HT material in this study was between 80 and 200 MPa (depending on the assumed intrinsic flaw size). The mismatch between the strength found by Entezari and that found in this study is substantially owed to the different architecture of the analyzed scaffolds. The microstructure of scaffolds in Ref.^36^ was a regular grid as that obtained in a robocasting technique which results in a normalized strength (i.e. macroscopic strength divided by the intrinsic tensile (flexural) strength) substantially higher than that obtained in foam-replicated structures. In particular, for porosity close to 50% a normalized strength of 0.44 was found for the regular grid; on the contrary, the foam replicated scaffold of this study at 56% has shown a normalized strength of 0.06. The regular arrangement of the robocast scaffolds subjected to a compressive force along a direction perpendicular to the planes of the fibers is such that the load is mainly carried out through compression at the fiber intersection; on the contrary, the random pore geometry of the foam replicated scaffolds induces, locally, tensile stress which promotes fracture propagation across the pores. In a study by Touaiher et al.^17^ the crack pattern generated under uniaxial compression in a hydroxyapatite scaffold was evaluated at different load steps through $\upmu$CT imaging. The microstructure was a regular grid and fractures propagate parallel to the load direction in the free portions of the rods. This behavior is consistent to what we found in a previous study^21^ where we applied the same algorithm of the present study for fracture prediction, matching the crack pattern of^17^. This comparison confirms how $\upmu$CT-based finite element models can be successfully used to predict the nucleation and evolution of cracks in brittle porous scaffolds.

Similar to what has been found for macroscopic elastic modulus, also the macroscopic normalized strength was substantially affected by the sintering temperature. The normalized strength of LT scaffolds is higher than that of HT scaffolds. This clearly shows that the two-scale porosity found in HT scaffold is detrimental to the macroscopic strength; the higher intrinsic strength of HT material is sufficient to obtain HT samples with slightly higher strength compared to LT scaffolds. Fracture propagation mechanisms are clearly affected by the small pores in HT samples as fractures propagate through the many micro-pores which act as stress concentrators. Although a direct comparison between the computational estimates and the experiments reported in Ref.^32^ was not possible as the $\upmu$CT images of the tested samples were not available, a comparison on a normalized strength ($\sigma _0^{EXP}$ and $\sigma _0^{FEM}$) value which approximately accounts for the sample porosity was used. The comparison in terms of normalized strength clearly indicates a significant trend of strength increase with sintering temperature, see Fig. 4. Similarly to what Fiume et al. discovered^32^, an increase in overall scaffold strength as the sintering temperature rises is consistent with their findings, showing that even though the HT scaffold’s architecture was unfavourable due to the presence of numerous small pores, the improved intrinsic strength of the solid material compensates for the effect of fracture caused by the micropores.

## Conclusions

The present study’s outcomes underscore the significance of comprehending the mechanical characteristics of the constituent materials and having a numerical tool that can accommodate manufacturing process peculiarities when designing and fabricating scaffolds that are biomechanically dependable for bone tissue engineering. In particular, in this work, a combination of effective computational models and Computed micro-Tomography shed light on the effect of the sintering temperature on the mechanical and morphological properties of glass-derived scaffolds. Additional information, not available in conventional laboratory testing, has also been disclosed by these techniques, such as the fracture pattern and how it is affected by the micro-scale morphology of the scaffolds sintered at different temperatures.

## Methods

### Scaffold preparation and computed micro-tomography

The scaffolds characterized in this project were obtained through the foam replication technique, following the process described extensively by Fiume et al.^31^.

The constituent material is an experimental ${\text {SiO}}_{2}$-based bioactive glass ($47.5{\text {SiO}}_{2}-10{\text {Na}}_{2}{\text {O}}-10{\text {K}}_{2}{\text {O}}-10{\text {MgO}}-20{\text {CaO}}_{2}-2.5{\text {P}}_{2}{\text {O}}_{5}$ mol%) referred to as 47.5B.

Six different temperatures, ranging from 600 to 850 $^{\circ }$C with a step increment of 50$^{\circ }$C, were used during the sintering process, in order to investigate the effects of temperature on the mechanical properties of the obtained scaffolds. One scaffold for each sintering temperature was analyzed.

The 3D geometrical representation of the micro-architecture of the scaffolds was obtained by post-processing the $\upmu$CT images. X-ray $\upmu$CT scanning of the glass derived scaffolds was performed in air by a Phoenix Nanotom S (Waygate Technologies/Baker Hughes Digital Solutions GmbH, Wunstorf, Germany). Projection images were collected using a source voltage of 110 kV and a source current of $110\;\upmu$A, with no X-ray filters employed. A voxel size of $5.00\;\upmu$m was achieved by using a 10x geometrical magnification. A power mode with maximum target X-ray emission power of 2.7 W was used with 1.5 s exposure time, $0.5^{\circ }$ step-size in rotation and integration of three images for each rotation step, thus obtaining 720 projections.

Virtual volumes were reconstructed from the projection images using the datos-x reconstruction software provided by the equipment manufacturer. Using the VGStudio Max 3.3 software (Volume Graphics, Heidelberg, Germany), 6 sets of 16-bit grey-scale images (pixel size $5\;\upmu$m) were prepared from the 3D data, one for each scaffold type.

Each image set was post-processed through MATLAB, Fiji-ImageJ and the BoneJ 7.0.11 plugin^37^. First, a Gaussian Blur filter (radius 2 voxels) was applied to the image stack; the images were then subjected to a resampling process in order to reduce the total number of voxels, getting a voxel size of $10\;\upmu$m. The binarization threshold was set by means of the Otsu algorithm^38^. A tilting process was carried out, based on the centroid axis inclination, in order to align the longitudinal axis of the cylindrical sample with the direction of the load (z axis). As the top and bottom parts of the scaffolds exhibited irregular shapes and asperities, a portion of the top and bottom layers were removed to ensure enough solid fraction at the boundaries.

### Morphological characterization

Scaffold porosity was obtained for all six samples. Each binary stack was subjected to a connectivity analysis with the purpose to get rid of the objects not connected to the main scaffold structure. The porosity was evaluated as the ratio between the number of empty voxels (0 value), excluding the voids surrounding the cylinders, and the total number of voxels.

Furthermore, local solid wall thickness and local pore size were determined by means of the BoneJ plugin for Fiji-ImageJ^37^. In general, the wall thickness at a point was obtained by determining the largest sphere diameter entirely contained in the wall’s solid structure. A similar definition was given for the pore size.

The BoneJ plugin also determined a mean value, maximum value and standard deviation based on the values of local wall thickness and pore size.

Based on the values obtained, histograms such as the ones reported in Fig. 2 were obtained; the two temperatures reported were chosen as representative, as they correspond to the lowest and highest sintering temperatures evaluated in this study.

The pore size data corresponding to 850 $^{\circ }$C for the HT group was fitted by using a bimodal distribution; its minimum was found to correspond to $128\;\upmu$m. This value was therefore identified as the threshold separating large pores, such as the ones found also in the LT group, and smaller pores, which appear with a significantly higher percentage in the HT group.

### Finite element models

The macroscopic elastic properties and compressive strength were estimated by means of the finite element method. The open-source multigrid finite element code ParOSol^39^ was used for this purpose. The ParOSol solver makes use of a voxel-based Cartesian finite element grid; in this work, one finite element was used for each voxel in the re-sampled $\upmu$CT images. Cubic elements and eight nodes with three-linear shape functions were used with a characteristic element size of $10\;\upmu$m. Six cylindrical models were analyzed, with approximate height and diameter of 5 mm and 8 mm, respectively. The models counted from 80 to 150 million of finite elements. A uniaxial compression load was simulated by applying a downward displacement on the top surface of the sample along the axial direction (z-direction). The axial displacement component at the bottom surface was constrained. Furthermore, rigid body motions were constrained by setting displacement constraints on two selected nodes on the outer boundary at the bottom surface. The elastic moduli of the six different constituent materials (one for each temperature) were those obtained experimentally in Ref.^33^ through nanoindentation experiments on bulk materials; six bulk samples were indented, in order to quantify the relationship between the sintering temperatures and the elastic moduli. Two different values for the tensile strength were obtained from the experimental measure of the critical stress intensity factors and assuming two different values of intrinsic initial flaw. Indeed, the classical fracture mechanics arguments indicate that the intrinsic flaw size determines the tensile strength of sintered ceramic materials; in the paper by D’Andrea et al.^33^, these arguments were used to evaluate the characteristic tensile strength of the glass-ceramic as a function of the flaw size. By selecting two representative values of flaw size ($10\;\upmu$m and $20\;\upmu$m), two distinct values of the intrinsic tensile strength were determined and consequently, a range of effective compressive strength of the scaffolds was found. All material parameters are reported in Table 3.

**Table 3:** Intrinsic mechanical properties of the materials^33^.

Sintering temperature [${}^\circ$C]	Elastic modulus [GPa]	Tensile strength [MPa]	
		d $= 10\,\upmu$m	d $= {20}\,\upmu$m
600	$79.97 \pm 2.36$	105	75
650	$84.4 \pm 1.11$	114	81
700	$101.7 \pm 2.51$	135	96
750	$103.01 \pm 4.25$	119	84
800	$102.81 \pm 3.07$	189	134
850	$102.39 \pm 4.27$	223	159

The simulation of the fracture propagation process upon compression load on the sample is carried out by using the sequential procedure as described in Ref.^40^ and used in Ref.^21^. Compressive strength is here assumed to be ten times higher than the tensile strength as found to be the case for many ceramic materials^41^. The macroscopic stress in the sample was obtained as the volumetric average of the local stress, while the macroscopic compressive strain was calculated as the ratio between the applied axial displacement and the total height of the samples. The macroscopic elastic moduli of the samples were obtained as the initial slope in the macroscopic stress-strain curve. While, the macroscopic compressive strength was estimated as the maximum stress achieved before a significant drop in the bearing capacity of the sample.

The experimental values of compressive strength were evaluated in a previous study^32^ through destructive crushing tests; scaffolds obtained with the same production method and all the six different sintering temperatures were tested. It was therefore possible to compare the computational values obtained in this study with experimental values present in literature^32^. In order to compare the computational and experimental values of compressive strength of the scaffolds exhibiting different porosity ($\phi$) and intrinsic strength ($\sigma _0$), both dependent on the sintering temperature, the following equation^34^

1 $$\begin{aligned} \sigma = \sigma _0 \cdot 0.2(1-\phi )^{3/2} \end{aligned}$$

was used to define two temperature-dependent parameters $\sigma _0^{FEM}$ and $\sigma _0^{EXP}$ that are independent of the scaffold porosity as:

2 $$\begin{aligned} \sigma _0^{FEM}=\frac{\sigma ^{FEM}}{0.2 \left( 1-\phi ^{FEM} \right) ^{3/2}}; \;\;\;\;\;\;\; \sigma _0^{EXP}=\frac{\sigma ^{EXP}}{0.2 \left( 1-\phi ^{EXP} \right) ^{3/2}} \end{aligned}$$

where $\sigma ^{FEM}$ and $\sigma ^{EXP}$ are the computational and experimental global strength of the scaffold, respectively, and $\phi ^{FEM}$ and $\phi ^{EXP}$ are the computational and experimental porosity of the scaffold, respectively.

The effect of small pores on the fracture propagation phenomenon was evaluated in micropores having a characteristic size smaller than $128\;\upmu$m, as mentioned in earlier sections. The evaluation of the micropores connected by the fracture path was carried out as follows:

3 $$\begin{aligned} \chi = \frac{V_{\chi }}{V} \cdot 100 \% \end{aligned}$$

where *V* represents the volume of voxels exhibiting a spacing less than $128\;\upmu$m and $V_{\chi }$ the volume of pores connected by the fractures.

### Ethical issues

In this study no experiments were performed on human or animal subjects.

## Data Availability

Data will be made available from the corresponding Authors upon reasonable request.
